# The ER/Golgi Protein FNDC3B Facilitates Climbing Fibre to Purkinje Cell Synapse Elimination in the Developing Mouse Cerebellum

**DOI:** 10.1111/ejn.70411

**Published:** 2026-01-23

**Authors:** Céline Louise Mercier, Takaki Watanabe, Yuto Okuno, Kyoko Matsuyama, Kyoko Kushibe, Henry Denny, Taisuke Miyazaki, Miwako Yamasaki, Meiko Kawamura, Manabu Abe, Kenji Sakimura, Masahiko Watanabe, Naofumi Uesaka, Masanobu Kano

**Affiliations:** ^1^ Department of Neurophysiology, Graduate School of Medicine The University of Tokyo Tokyo Japan; ^2^ International Research Center for Neurointelligence (WPI‐IRCN), The University of Tokyo Institutes for Advanced Study (UTIAS), The University of Tokyo Tokyo Japan; ^3^ Advanced Comprehensive Research Organization (ACRO), Teikyo University Tokyo Japan; ^4^ Department of Anatomy, Faculty of Medicine Hokkaido University Sapporo Japan; ^5^ Department of Animal Model Development, Brain Research Institute Niigata University Niigata Japan; ^6^ Department of Cognitive Neurobiology, Graduate School of Medical and Dental Sciences Institute of Science Tokyo Tokyo Japan

**Keywords:** cerebellum, climbing fiber, FNDC3B, postnatal development, Purkinje cell, synapse elimination

## Abstract

Synapse elimination during development is crucial for refining neural circuits by removing excess synapses formed around birth. In the neonatal cerebellum, Purkinje cells (PCs) are initially innervated by multiple climbing fibers (CFs) with similar synaptic strengths. During subsequent postnatal development, a single CF is strengthened and retained, while the other CFs are eliminated. Here, our PC‐specific RNAi knockdown (KD) screening revealed that fibronectin type III domain containing 3B (FNDC3B), an endoplasmic reticulum protein, was involved in CF synapse elimination from around postnatal day 9 (P9) in mice. We showed that FNDC3B mRNA was expressed in PCs during CF synapse elimination. In PC‐selective FNDC3B conditional knockout (FNDC3B‐cKO) mice, CF synapse elimination from P10 was impaired, and the extension of CFs along PC dendrites was reduced at P21. However, these phenotypes were recovered by P40. In contrast, parallel fiber‐mediated excitatory synaptic inputs and inhibitory synaptic inputs to PCs were not affected in FNDC3B‐cKO mice. These results suggest that FNDC3B facilitates CF synapse elimination during postnatal development, highlighting a new role of FNDC3B in the developing brain.

## Graphical abstract

We investigated the role of fibronectin type III domain containing 3B (FNDC3B) in climbing fiber (CF) to Purkinje cell (PC) synapse elimination during postnatal development of the mouse cerebellum. We performed PC‐specific deletion of FNDC3B by RNAi‐mediated knockdown or PC‐specific conditional knockout in mice and examined CF to PC synapse development both electrophysiologically and morphologically. Our results show that FNDC3B in PCs facilitates CF synapse elimination from around postnatal day 10 and the extension of CFs along PC dendrites, but the effects of FNDC3B deletion in PCs are recovered in the adult cerebellum.

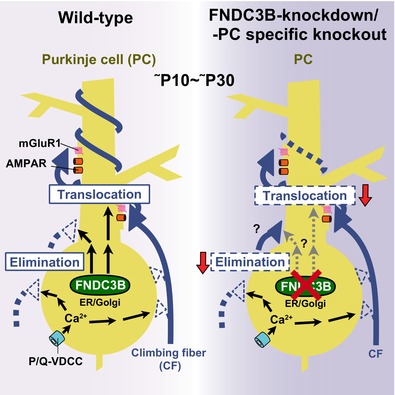

**Abbreviations**
ACSF: artificial cerebrospinal fluid
Arc: activity‐regulated cytoskeleton‐associated protein
Bai3: brain‐specific angiogenesis inhibitor 3
BDNF: brain‐derived neurotrophic factor
C1ql1: C1q‐like molecule 1
CaMKIIα: Ca^2+^/calmodulin‐dependent protein kinase II α
CF: climbing fiber
cKO: conditional knockout
EGFP: enhanced green fluorescent protein
EGL: external granular layer
EPSC: excitatory postsynaptic current
ER: endoplasmic reticulum
FAD104: factor for adipocyte differentiation‐104
FISH: fluorescent in situ hybridization
FNDC3B: fibronectin type III domain containing 3B
GAD67: 67 kDa glutamic acid decarboxylase
IGL: internal granular layer
KD: knockdown
mGluR1: type 1 metabotropic glutamate receptor
mIPSC: miniature inhibitory postsynaptic current
ML: molecular layer
P/Q‐VDCC: P/Q‐type voltage‐dependent Ca^2+^ channel
PC: Purkinje cell
PCL: Purkinje cell layer
PF: parallel fiber
PKCγ: protein kinase Cγ
PLCβ: phospholipase Cβ
PlxnA4: plexin A4
PPR: paired‐pulse ratio
Sema3A: semaphorin 3A
Sema7A: semaphorin 7A
STAT3: signal transducer and activator of transcription 3
VGluT: vesicular glutamate transporter
VIAAT: vesicular inhibitory amino acid transporter
ZFP64: zinc finger protein 64

## 1 Introduction

Synapse elimination is a fundamental developmental process that shapes functionally mature neural circuits during postnatal development. In the nervous systems of neonatal animals, massive synaptogenesis occurs, and redundant synaptic connections are initially formed. Subsequently, some synaptic connections are strengthened and retained, and others are weakened and eventually eliminated (Katz and Shatz 1996; Lichtman and Colman 2000; Riccomagno and Kolodkin 2015). As a representative model of synapse elimination, the postnatal development of climbing fiber (CF) to Purkinje cell (PC) synapses in the rodent cerebellum is widely known (Watanabe and Kano 2011; Kano et al. 2018; Kano and Watanabe 2019). PCs of the neonatal mouse cerebellum receive excitatory synaptic inputs with similar strengths from more than four CFs that originate from neurons in the contralateral inferior olive. Among multiple CFs innervating the soma of each PC, synaptic inputs from a single CF become progressively stronger relative to those from the other CFs until around postnatal day 7 (P7) (termed “functional differentiation”), and the strongest CF expands its synaptic territory along the growing primary dendrites of the PC from around P9 (termed “CF translocation”). Synapses from the other weaker CFs remaining on the soma are eliminated in two distinct processes from around P7 to around P11 (termed “early phase of CF elimination”) and from around P12 to around P17 (termed “late phase of CF elimination”) (Kano et al. 2018).

Previous studies show that the activity of PCs is a crucial factor for CF synapse elimination (Andjus et al. 2003; Lorenzetto et al. 2009), and that two canonical signaling pathways in PCs triggered by the activation of type 1 metabotropic glutamate receptor (mGluR1) (Kano et al. 1997; Ichise et al. 2000) and P/Q‐type voltage‐dependent Ca^2+^ channel (P/Q‐VDCC) (Miyazaki et al. 2004; Hashimoto et al. 2011) are essential for the multiple processes of CF synapse elimination (Kano et al. 2018; Kano and Watanabe 2019). mGluR1 and its downstream signaling cascade in PCs, consisting of Gαq (Offermanns et al. 1997), phospholipase Cβ3 and β4 (PLCβ3, PLCβ4) (Kano et al. 1998; Rai et al. 2021), and protein kinase Cγ (PKCγ) (Kano et al. 1995), are involved mainly in the late phase of CF elimination. Proper formation of parallel fiber (PF) to PC synapses is a prerequisite for the late phase of CF elimination (Hashimoto, Yoshida, et al. 2009), and the neural activity along the mossy fiber‐granule cell‐PF circuit is considered to activate mGluR1 at PC dendrites (Nakayama et al. 2024). The mGluR1 signaling in PCs leads to the production of Semaphorin 7A (Sema7A) (Uesaka et al. 2014) and brain‐derived neurotrophic factor (BDNF) (Choo et al. 2017) that retrogradely affect weaker CFs to propel their elimination. Moreover, the immediate early gene *Arc/Arg3.1* also facilitates the removal of redundant CF synapses from the PC soma during the late phase of CF elimination (Mikuni et al. 2013), which is activated by Ca^2+^ influx into PCs through P/Q‐VDCC (Mikuni et al. 2013; Kawata et al. 2014). By contrast, the early phase of CF elimination is independent of PF‐PC synapse formation (Hashimoto et al. 2001; Hashimoto, Yoshida, et al. 2009), but it is critically dependent on CF synaptic inputs that activate P/Q‐VDCCs and induce Ca^2+^ entry into PCs (Hashimoto et al. 2011). P/Q‐VDCC is also crucial for selecting a single “winner” CF and the dendritic translocation of the winner CF (Hashimoto et al. 2011). Moreover, proper intensity of GABAergic inhibition of PCs to control P/Q‐VDCC‐mediated Ca^2+^ entry into PCs is required for CF synapse elimination from around P10 (Nakayama et al. 2012; Lai et al. 2025). However, relatively little is known about the molecular mechanisms of the early phase of CF elimination.

Searching for molecules involved in CF synapse elimination, we performed RNAi‐mediated knockdown (KD) screening of transmembrane proteins expressed in developing PCs (Uesaka et al. 2014). Among several candidate molecules identified in the screening, we focused on FNDC3B (fibronectin type III domain containing 3B) because this molecule showed no similarity to any of the molecules previously known to be involved in CF synapse elimination. FNDC3B, also known as FAD104 (factor for adipocyte differentiation‐104), contains nine repeats of fibronectin type III domains and a transmembrane domain in the C‐terminus (Tominaga et al. 2004), and has been suggested to localize to the endoplasmic reticulum (ER) (Nishizuka et al. 2009; Fucci et al. 2020) or Golgi apparatus (Cai et al. 2012) in in vitro culture cells. FNDC3B has been shown to play an essential role in adipogenesis, embryonic bone formation, and lung maturation, resulting in rapid neonatal death in FNDC3B‐deficient mice (Tominaga et al. 2004; Nishizuka et al. 2009; Kishimoto et al. 2011, 2013). Several studies have reported that the aberrant expression of FNDC3B has been observed in various cancers, acting as a potential biomarker (Katoh et al. 2015; Fucci et al. 2020; Han et al. 2020), as well as in the brain tissue of patients with epilepsy and brain tumors (Kwon et al. 2023). However, the physiological function of FNDC3B in the brain is still largely unknown.

In the present study, we first scrutinized the effect of RNAi‐mediated KD of FNDC3B in PCs on CF innervation of PCs during postnatal development. We then generated PC‐specific FNDC3B conditional knockout (FNDC3B‐cKO) mice in which we examined CF innervation of PCs both electrophysiologically and morphologically during postnatal development and in maturity, as well as PF‐PC synapses. Our data demonstrate that FNDC3B in PCs promotes CF elimination from the early phase and CF dendritic translocation without affecting the selection of a single winner CF and PF‐PC synapse formation and function.

## 2 Materials and Methods

### 2.1 Animals

C57BL/6N wild‐type mice of both sexes were used for lentivirus‐mediated knockdown experiments (SLC Japan). FNDC3B‐floxed mice are generated in C57BL/6N background by inserting the loxP sequence into both sides of exon 6 (244 nt) of *Fndc3b* genome (FNDC3B transcript: ENSMUST00000195008.6). Deletion of exon 6 by Cre recombinase results in a frameshift that immediately induces a stop codon in exon 7. To selectively express Cre in PCs, heterozygous male GluD2‐Cre mice (Hashimoto et al. 2011) were crossed with homozygous female FNDC3B‐floxed mice to finally get homozygous FNDC3B‐floxed; GluD2‐Cre mice (FNDC3B‐cKO) and homozygous FNDC3B‐floxed mice without Cre as a control. Male and female mice were randomly chosen for all experiments. All experiments were approved by the institutional animal care and use committee and were conducted following the guidelines of the University of Tokyo, Hokkaido University, Niigata University, and Japan Neuroscience Society.

### 2.2 Viral Vector Constructs

Vesicular stomatitis virus G (VSV‐G) pseudotyped lentiviral vectors (pCL20c) were constructed as previously described (Uesaka et al. 2012) and were designed under the control of a truncated L7 promoter (pCL20c‐trL7) for Purkinje cell‐specific expression. The BLOCK‐iT Pol II miR RNAi Expression Vector Kit (Invitrogen) and the BLOCK‐iT RNAi designer were used for engineered miRNA with the following sequences:

5′‐TGCTGATAACCTGGAGGCACATGGATGTTTTGGCCACTGACTGACATCCATGTCTCCAGGTTAT—3′ and 5′—CCTGATAACCTGGAGACATGGATGTCAGTCAGTGGCCAAAACATCCATGTGCCTCCAGGTTATC—3′ for the miRNA against FNDC3B;

5′‐GCTGTTCCAATGAAGTATGGTTCCGGTTTTGGCCACTGACTGACCGGAACCACTTCATTGGAA‐3′ and 5′‐CCTGTTCCAATGAAGTGGTTCCGGTCAGTCAGTGGCCAAAACCGGAACCATACTTCATTGGAAC‐3′ for P/Q‐VDCC miRNA;

The oligonucleotides were then subcloned into pCL20c‐trL7 vector at the 5′‐side of enhanced green fluorescent protein (EGFP) or mOrange. The cDNA for FNDC3B was obtained by RT‐PCR of a cDNA library from the mouse cerebellum at P7. The following PCR primers were used:

5′‐ATGTACGTCACCATGATGATGACCG‐3′ and 5′‐TTACTTCATTAAGAAGTACTGTAATATAAAGGCAAACAAAATGG‐3′.

We generated an RNAi‐resistant form of FNDC3B (FNDC3B‐RES) using the QuikChange Lightning site‐directed mutagenesis kit (Agilent Technologies). FNDC3B‐RES and mOrange2 were linked in‐frame, interposed by picornavirus “self‐cleaving” P2A peptide sequence to enable efficient bicistronic expression, and then subcloned into pCL20c‐trL7. The following sequences were used to generate FNDC3B‐RES:

5′‐ ACCCAATGGGTCCATTCCTCCTATACACGTACCCCCGGGATACATCTCACAGGTGATTGAAGACAG‐3′ and 5′‐ CTGTCTTCAATCACCTGTGAGATGTATCCCGGGGGTACGTGTATAGGAGGAATGGACCCATTGGGT‐3′.

FNDC3B‐RES was constructed by altering the last nucleotide of each codon from the target sequence of FNDC3B miRNA so that each codon coding the same amino acid might not be recognized by the miRNA. The cDNA constructs were verified by DNA sequencing.

### 2.3 Lentivirus Preparation

The lentivirus was produced by transfection of sub‐confluent Human Embryonic Kidney (HEK) 293T cells with a mixture of two packaging plasmids (7 μg of psPAX2 and 3.5 μg of pCAG‐VSV‐G) and a lentivirus vector (10 μg of pCL20c‐tr7‐FNDC3B‐miRNA‐GFP (FNDC3B‐KD), 20 μg of pCL20c‐tr7‐FNDC3B‐RES‐mOrange2 (FNDC3B‐RES)) as described previously (Uesaka et al. 2012). Cells were cultured in DMEM (Invitrogen) supplemented with 10% fetal bovine serum (Hyclone), 100 U/mL penicillin G, and 0.1 mg/mL streptomycin. Cells were incubated at 37°C, pH 7.35, in a 5% CO_2_ atmosphere. The cultured medium was exchanged with fresh medium 24 h after the transfection. Then, 48 h after transfection, the medium containing the virus vector particles was harvested. The medium was filtered through 0.22 μm membranes and centrifuged at 27,000 rpm for 90 min. The lentivirus samples were suspended in 30 to 35 μL of phosphate buffer saline (PBS) and stored at −80°C until lentivirus injection.

### 2.4 Lentiviral Injection In Vivo

C57BL/6N mice at P1–2 were used for in vivo virus infection (Okuno et al. 2023; Zhang et al. 2025). A mouse was anesthetized by inhalation of isoflurane (1%–3%) and was attached to a head‐holding device. Then, a sagittal incision was made to expose the cranium over the cerebellar vermis, and a hole was drilled at the midline. A 33‐Gauge Hamilton syringe filled with viral solution was attached to a micropump (UltramicroPump II, World Precision Instruments (WPI)) and placed on the surface of the cerebellar vermis through the hole. 3.5 μL of the viral solution was injected at a rate of 200 nL/min using a microprocessor‐based controller (Micro4, WPI). The syringe was left in place for 3 min after the injection before it was withdrawn. The incision was then sutured, and the mouse was returned to its home cage.

### 2.5 Evaluation of Knockdown Efficacy

HEK293T cells in a 24‐well dish were transfected using X‐tremeGENE 9 reagents (Roche) with one of the following vector combinations: GFP and FNDC3B‐cDNA expression vectors for the control, FNDC3B‐KD and FNDC3B‐cDNA expression vectors for the KD test, or FNDC3B‐KD and RNAi‐resistant FNDC3B‐RES expression vectors for the rescue test. At first, 0.5 μg of the FNDC3B miRNA knockdown vector and the control vector were transfected into different wells. Six hours later, 0.2 μg of the same vectors, with 0.3 μg mOrange2‐linking FNDC3B cDNA/RES in each well, were transfected. Forty‐eight hours after the last transfection, the cells were fixed with 4% Paraformaldehyde (PFA) in 0.1 M phosphate buffer for 10 min at room temperature. After washing with PBS, the cells were incubated in 0.5% Triton X‐100/PBS for 30 min, thereafter incubated with 10% donkey serum for 1 h at room temperature, and washed with PBS. The cells were incubated overnight at 4°C in 0.1% Triton X‐100/PBS with primary antibodies: anti‐GFP (rat, 1:1000, Nacalai Tesque) and anti‐FNDC3B (rabbit, 1:500, Atlas antibodies#HPA007859). After washing with PBS, the following secondary antibodies were added: anti‐rat Alexa Fluor 488 (1:300, Jackson ImmunoResearch) and anti‐rabbit Cy3 (1:300, Jackson ImmunoResearch). Fluorescence signals were observed under a confocal laser‐scanning microscope (FV1200, Evident) and analyzed using Fiji/ImageJ (National Institutes of Health).

### 2.6 Electrophysiology

Mice from P8 to P42 were anesthetized with CO_2_ and immediately decapitated. Brains were removed and immersed in a chilled (0°C–4°C) artificial cerebrospinal fluid (ACSF) composed of 125 mM NaCl, 2.5 mM KCl, 2 mM CaCl_2_, 1 mM MgSO_4_, 1.25 mM NaH_2_PO_4_, 26 mM NaHCO_3_, and 20 mM glucose bubbled with 95% O_2_ and 5% CO_2_. Parasagittal cerebellar slices (250 μm in thickness) were prepared using a vibratome slicer (VT1200S, Leica). The slices were then placed in a reservoir chamber and bathed in oxygenated ACSF at room temperature for 1 h for recovery. The recording chamber, located on the stage of an upright microscope (Olympus), was continuously perfused with oxygenated ACSF at 32°C. Whole‐cell recordings were conducted from the soma of visually identified PCs (Edwards et al. 1989). For recording CF‐mediated excitatory postsynaptic currents (CF‐EPSCs) and PF‐mediated EPSCs (PF‐EPSCs), picrotoxin (100 μM, Nacalai) was added to the bath solution. For recording miniature inhibitory postsynaptic currents (mIPSCs), NBQX (10 μM, Tocris), AP‐5 (50 μM, Tocris), and tetrodotoxin (1 μM, Nacalai) were added to the bath solution. The patch pipettes for recording CF‐EPSCs and PF‐EPSCs were filled with an intracellular solution containing 60 mM CsCl, 10 mM Cs D‐Gluconate, 20 mM TEA‐Cl, 20 mM BAPTA, 4 mM MgCl_2_, 4 mM Na_2_‐ATP, and Na_2_‐GTP (pH 7.3 adjusted with CsOH). The patch pipettes for recording mIPSCs were filled with an intracellular solution containing 124 mM CsCl, 10 mM HEPES, 10 mM BAPTA, 1 mM CaCl_2_, 4.6 mM MgCl_2_, 4 mM ATP, 0.4 mM GTP (pH 7.3 adjusted with CsOH). The resistance of the patch pipettes was 1.7–2.5 MΩ, and the pipette access resistance was compensated by 70%. CFs were stimulated (duration, 0.1 ms; interval, 50 ms; current intensity, 0–100 μA) in the granular layer 20 to 100 μm away from the PC soma through a pipette filled with ACSF at the holding potential of −10 mV. To stimulate all CFs innervating the PC under recording, the stimulating pipette was systematically moved around the PC soma under recording, and the stimulus strength was gradually increased from 0 μA to a maximum of 100 μA at each stimulation site. PFs were stimulated with a glass pipette filled with ACSF in the middle of the molecular layer. The position of PF stimulation was adjusted to the site where stimulation with 10 μA current intensity elicited the maximal PF‐EPSCs without contamination by CF‐EPSCs at the holding potential of −70 mV. To obtain the input–output relation of PF‐EPSCs, the stimulus intensity was decreased from 10 to 1 μA. For recording mIPSCs, recording was started 3 min after establishing the whole‐cell recording configuration at the holding potential of −70 mV. Ionic currents were recorded with an EPC 9/10 patch‐clamp amplifier (HEKA Electronik) or a Double Integrated Patch Amplifiers (Sutter Instrument). The signals were digitized at 20 kHz and filtered at 1–3 kHz. Online data acquisition and offline data analysis were performed using PatchMaster (HEKA‐Electronik), Igor Pro 9 (Wave Metrics), and MATLAB (MathWorks) software.

In experiments on FNDC3B‐KD mice, EGFP‐positive PCs with FNDC3B‐KD and EGFP‐negative control PCs were recorded in the same slices to evaluate the effects of FNDC3B‐KD. In experiments on mice expressing the RNAi‐resistant form of FNDC3B, EGFP and mOrange2 double‐positive PCs, which expressed both the miRNA against FNDC3B and cDNA for the RNAi‐resistant form of *Fndc3b*, and the non‐fluorescent control PCs were recorded in the same slices. For all electrophysiological experiments comparing FNDC3B‐Ctrl and FNDC3B‐cKO mice, the genotypes were verified by an independent individual. The experimenters then performed data acquisition, analysis, and interpretation in a fully blinded manner until all analyses were completed.

### 2.7 Quantification of Disparity in Multiple CF‐EPSCs

To quantify the disparity in multiple CF‐EPSCs recorded in each PC, the disparity index and disparity ratio were calculated (Hashimoto and Kano 2003). To calculate the disparity ratio, the amplitudes of individual CF‐EPSCs in a given multiply‐innervated PC were measured, and they were numbered in the order of their amplitudes (A_1_, A_2_, .., A_N_, *N* ≥ 2; N is the number of climbing fibers innervating a given PC. A_N_ represents the largest CF‐EPSC). The disparity ratio and the disparity index were represented by the following formulas:

$$\text{Disparity ratio}=\frac{\left(\frac{A_{1}}{A_{N}}+\frac{A_{2}}{A_{N}}+\cdots +\frac{A_{N-1}}{A_{N}}\right)}{\left(N-1\right)}$$

$$\text{Disparity index}=\frac{S.D.}{M}$$

$$M=\sum \frac{A_{i}}{N}\;\left(i=1,2,\ldots N;N\geq 2\right)$$

$$S.D.=\sqrt{\sum \frac{{\left(A_{i}-M\right)}^{2}}{N-1}}$$

*A*_*i*_ are the CF‐EPSC amplitudes recorded at the same holding potential. The disparity ratio represents the average of the inverse proportion of the strongest CF‐EPSC amplitude to each of the weaker CF‐EPSCs. Therefore, if the differences between *A*_*N*_ and the amplitudes of smaller CF‐EPSCs are large, the disparity ratio will be small. If CF‐EPSCs have similar amplitudes, the disparity ratio will be close to one. The disparity index is the coefficient of variation for all CF‐EPSC amplitudes. Hence, if CF‐EPSCs are variable in size, the value will be large. Conversely, if the amplitude of CF‐EPSCs is identical, the value of the disparity index will be small.

### 2.8 Fluorescent In Situ Hybridization (FISH)

Double FISH was performed using a fluorescein‐labeled cRNA probe for FNDC3B mRNA (nucleotides 188–3811; GenBank accession number NM_001356953.1), and a digoxigenin (DIG)‐labeled probe for 67 kDa glutamic acid decarboxylase (GAD67; nucleotides 1036–2015, GenBank accession number NM_008077). cRNA probes were synthesized by in vitro transcription using the pBluescript II plasmid vector encoding the above cDNAs as described previously (Yamasaki et al. 2010). Fresh brains were isolated from mice after anesthesia with isoflurane (5%, Pfizer, Manhattan, NY) and frozen in powdered dry ice. Fresh frozen sections were air‐dried and fixed by immersion in 4% paraformaldehyde (PFA) in 0.1 M phosphate buffer for 15 min. Sections (20 μm thick) were cut on a cryostat, and a set of sections from four postnatal stages (P7, P9, P12, and P15) was mounted on the same glass slide to be subjected to hybridization under the same conditions. In brief, sections were hybridized with a mixture of cRNA probes at a dilution of 1:1000. After post‐hybridization washes, each reporter molecule was visualized separately. First, fluorescein was detected using a peroxidase‐conjugated anti‐fluorescein antibody (Roche Diagnostics, 1:500) and the FITC‐TSA plus amplification kit (Akoya Biosciences, Marlborough, MA). Second, DIG was visualized with peroxidase‐conjugated anti‐DIG antibody (Roche Diagnostics, 1:500) and the Cy3‐TSA plus amplification kit (Akoya Biosciences). Sections were further stained with NeuroTrace 640/660 Nissl stain (Thermo Fisher Scientific). These fluorescent signals were taken with a confocal laser‐scanning microscope (FV1200, Evident, Tokyo, Japan). Expression levels of mRNA were evaluated semi‐quantitatively from confocal images taken with the same gain‐level settings. Separate colour components were converted to grayscale, and the grey level (arbitrary units) and area were measured using MetaMorph software (Molecular Devices, Downingtown, PA) from individual somata of GAD67‐positive PCs.

### 2.9 Western Blot Analysis

Cerebella from control and FNDCB‐cKO mice at P11 were collected, frozen in liquid nitrogen, and stored at −80°C until use. Samples were homogenized in RIPA buffer (Nacalai Tesque), and protein concentrations were measured using the Pierce 660 nm Protein Assay Reagent (Thermo Fisher Scientific). For SDS–PAGE, 15 and 1.66 μg of protein for detecting FNDC3B and β‐actin, respectively, were loaded onto a 10% polyacrylamide gel. Following electrophoresis, proteins were transferred to PVDF membranes and blocked with BLOCKING ONE (Nacalai Tesque). Primary antibodies (rabbit anti‐FNDC3B, 1:2000, Atlas Antibodies #HPA007859; mouse anti‐β‐actin, 1:10000, Sigma #A5316) were diluted in Solution A of Signal Enhancer HIKARI (Nacalai Tesque) and incubated with the membranes overnight at 4°C. After washing with PBS, the membranes were incubated with horseradish peroxidase–conjugated secondary antibodies (anti‐rabbit IgG, 1:1000; anti‐mouse IgG, 1:10000; Jackson ImmunoResearch), diluted in Solution B of Signal Enhancer HIKARI, for 1 h at room temperature. Chemiluminescent signals were developed using Chemi‐Lumi One L (Nacalai Tesque), and bands were visualized with an ImageQuant LAS 4010 system (GE Healthcare). Band intensities were quantified using ImageQuant TL software (GE Healthcare).

### 2.10 Immunohistochemistry

Under deep pentobarbital anesthesia (100 mg/kg body weight, i.p.), mice were transcardially perfused with 4% PFA in 0.1 M phosphate buffer and processed to obtain parasagittal microsliced sections (50 μm in thickness). After permeabilization with 0.1% Triton X‐100 in PBS and blocking with 10% normal donkey serum (Jackson ImmunoResearch, PA, USA) for nonspecific binding, cerebellar sections in 0.1% TritonX‐100/PBS were incubated at 4°C overnight with a mixture of primary antibodies (1 μg/mL) raised against the following molecules: anti‐calbindin (RRID AB_2571678), anti‐Car8 (RRID:AB_2571669), anti‐vesicular glutamate transporters VGluT1 (RRID AB_2571616) and VGluT2 (RRID AB_2571620), and anti‐vesicular inhibitory amino acid transporter (VIAAT; RRID AB_2571622). Then, a mixture of indocarbocyanin (Cy3) and Alexa Fluor 647‐labeled species‐specific secondary antibodies (1:200, Jackson ImmunoResearch, PA, USA; Thermo Fisher Scientific) was applied for 2 h at room temperature. Confocal images were taken with a laser‐scanning microscope (FV1200, Evident). Image analysis was performed using MetaMorph (Molecular Devices) or ImageJ (NIH).

### 2.11 Statistical Analysis

All statistical data are presented as mean ± standard error of the mean (SEM). Because CF‐EPSC step numbers are discrete, limited in range (1, 2, 3, and ≥ 4), and nonnormally distributed, the Mann–Whitney *U* test was used for two‐group comparisons, and the Kruskal–Wallis test, followed by the Steel–Dwass post hoc test, was applied for comparisons among three groups. For other datasets, normality was assessed using the Shapiro–Wilk test. Parametric or nonparametric statistical tests were applied to data with normal or nonnormal distributions, respectively. For two‐group comparisons, the Student's *t* test was used for CF‐EPSC kinetics, PPR of PF‐EPSC, and immunohistochemical data for vGluT1 and VIAAT, whereas the Mann–Whitney *U* test was used for immunohistochemical data for vGluT2, and for the amplitude and frequency of mIPSCs. For multiple‐group comparisons, two‐way repeated measures ANOVA with Tukey's post hoc test was applied for the analysis of PF‐EPSC input–output relations, whereas the Kruskal–Wallis test, followed by the post hoc Steel–Dwass test, was applied to evaluate KD efficacies and to quantify FISH signals. All statistical analyses were performed using EZR software (Kanda 2013) and GraphPad Prism software (GraphPad software). Differences between data sets were considered significant when *p* values were less than 0.05. “*”, “**”, “***” and ns represent *p* < 0.05, *p* < 0.01, *p* < 0.001 and not significant, respectively.

## 3 Results

### 3.1 Impaired CF Synapse Elimination in PCs With FNDC3B‐KD

**FIGURE 1:**
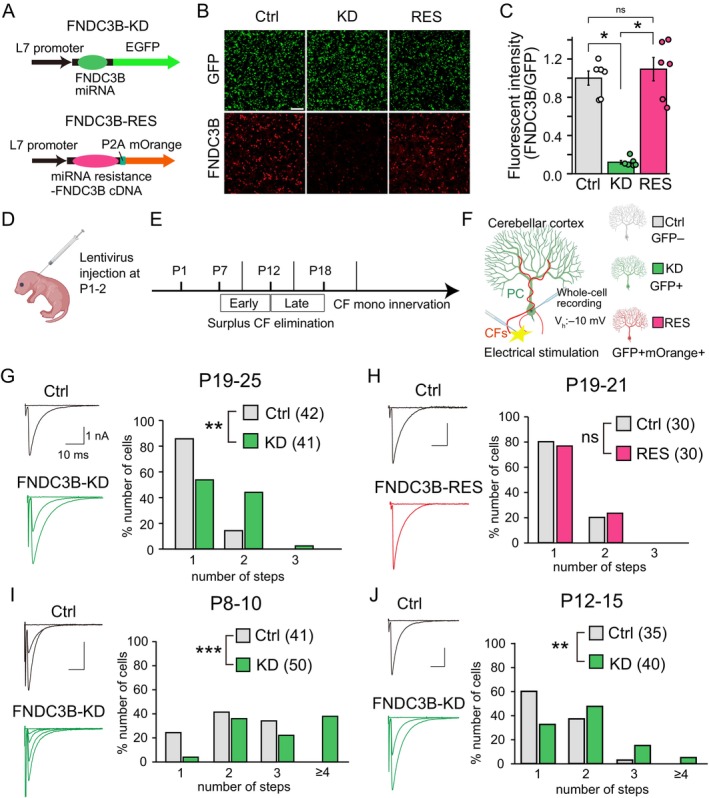
FNDC3B‐KD in PCs impairs CF synapse elimination. (A) Lentivirus vector constructs. The PC‐specific L7 promoter drives the expression of FNDC3B miRNA with GFP (FNDC3B‐KD) and miRNA‐resistant FNDC3B with mOrange2 (FNDC3B‐RES), respectively, in PCs. (B) Double immunostaining for EGFP (green) and FNDC3B (red) in HEK293T cells transfected with the FNDC3B + EGFP (left), FNDC3B + KD (middle), RES + KD (right). Scale bar, 100 μm. (C) Quantification of the fluorescence intensity of FNDC3B relative to EGFP in Ctrl (grey), KD (green), and RES (magenta) HEK293T cells (*n* = 6 wells, each). Kruskal‐Wallis test, *p* < 0.001, followed by Steel‐Dwass test, *p* = 0.01 (Ctrl vs. KD, KD vs. RES), and *p* = 0.702 (Ctrl vs. RES). (D) Virus injection into the cerebellum at P1–2. (E) Time course of CF‐PC synapse development showing the time points of electrophysiological experiments for G to J. (F) Whole‐cell recordings of CF‐EPSCs from PCs by electrical stimulation around the recorded PC in acute cerebellar slices. Ctrl, GFP− (grey); KD, GFP+ (green); RES, GFP+/mOrange+ (magenta). (G—J) CF innervation of PCs during P19–25 (G, 17 mice), P19–21 (H, 8 mice), P8–10 (I, 12 mice), and P12–15 (J, 16 mice). (left) Representative traces of CF‐EPSCs recorded from a Ctrl and a KD PC (G, I, J) or a control and a RES PC (H). Holding potential, −10 mV. Scale bars: 10 ms and 1 nA. (right) Frequency distribution histograms showing the number of CFs innervating each PC. Sample numbers of PCs are shown in parentheses. Mann–Whitney *U* test, *p* = 0.001 (G), *p* = 0.764 (H), *p* < 0.001 (I), and *p* = 0.008 (J).

We performed lentivirus‐mediated PC‐specific knockdown (KD) of FNDC3B in mice (Uesaka et al. 2012, 2014), using a lentiviral vector with EGFP and a miRNA against FNDC3B (FNDC3B‐KD) under the control of the L7 promoter (Figure 1A). The efficacy of FNDC3B‐KD was confirmed in HEK293T cells, showing effective suppression of FNDC3B expression to about 10% of the control level (Figure 1B,C). We injected the lentivirus for PC‐specific FNDC3B‐KD into the mouse cerebellum at P1–2, and then examined the CF innervation patterns of PCs at several developmental stages (Figure 1D–G). We prepared cerebellar slices from mice at respective ages, conducted whole‐cell recordings from GFP‐positive FNDC3B‐KD PCs (GFP+, KD) and GFP‐negative control PCs (GFP−, control) in the same slices, and recorded CF‐EPSCs (Figure 1D–F). CFs innervating the PC under recording were stimulated through a glass patch pipette filled with the external saline in the granule cell layer (Figure 1F). CF‐EPSCs were elicited with discrete steps, and the number of CFs innervating each PC could be estimated from the number of discrete CF‐EPSC steps elicited in that PC (Kano et al. 1995). Therefore, to stimulate all CFs innervating individual PCs, we moved the stimulation pipette systematically around the PC soma, and at each position, we increased the stimulus current intensity gradually from 0 to 100 μA. We counted the number of discrete CF‐EPSC steps and estimated the number of CFs innervating the PC under recording (Lai et al. 2025; Zhang et al. 2025). We found that the percentage of PCs innervated by more than one CF was significantly higher in FNDC3B‐KD PCs than in control PCs at P19–25 (Figure 1G), with the ratio of mono CF innervation being higher than 80% in control PCs but lower than 60% in FNDC3B‐KD PCs (Figure 1G). To exclude the possibility of off‐target effects of miRNA, we injected the lentivirus carrying both the FNDC3B‐KD vector and the FNDC3B‐RES vector for expressing the miRNA‐resistant form of FNDC3B and mOrange2 (Figure 1A,F,H). The degree of multiple CF innervation at P19–21 was similar between control PCs and PCs co‐expressing FNDC3B‐KD and the rescue construct (FNDC3B‐RES) (Figure 1H), excluding the possibility of off‐target effects of FNDC3B‐KD. These results suggest that FNDC3B in PCs is required for CF synapse elimination until around P20. Since the elimination of surplus CFs proceeds in two distinct developmental stages, namely the early phase (around P7 to around P11) and the late phase (around P12 to around P17) (Figure 1E) (Watanabe and Kano 2011; Kano et al. 2018), we next investigated at what developmental stage FNDC3B played a role in CF synapse elimination. We found that the degree of multiple CF innervation was higher in FNDC3B‐KD PCs than in control PCs during P8 to P10 (Figure 1I) and P12 to P15 (Figure 1J), indicating that FNDC3B in PCs is involved in the early phase of CF elimination.

### 3.2 FNDC3B Is Expressed in PCs During CF Synapse Elimination

**FIGURE 2:**
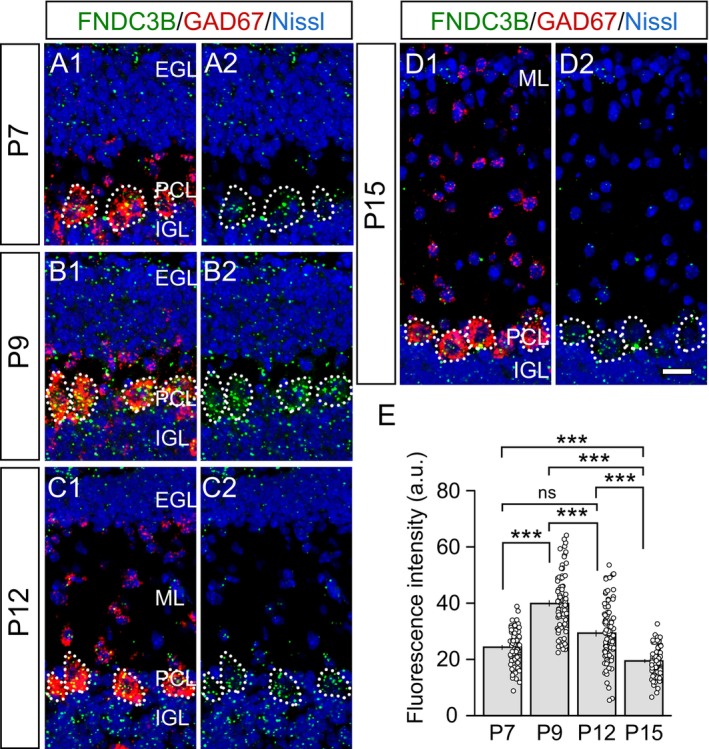
FNDC3B mRNA is expressed in PCs during postnatal development. (A–D) Double fluorescent in situ hybridization on parasagittal sections of the mouse cerebellum, depicting mRNAs for FNDC3B (green) and GAD67 (red) and counter‐stained signals for Nissl (blue), at P7 (A), P9 (B), P12 (C), and P15 (D). Dotted lines outline PC somata. EGL, external granular layer; ML, molecular layer; PCL, Purkinje cell layer; IGL, internal granular layer. Scale bar, 20 μm. (E) Summary bar graph showing the FNDC3B mRNA fluorescence intensity at P7, P9, P12, and P15 (*n* = 68, 91, 95, and 71 PCs, respectively). Kruskal–Wallis test, *p* < 0.001, followed by Steel‐Dwass test, *p* < 0.001 (P7 vs. P9), *p* = 0.079 (P7 vs. P12), *p* < 0.001 (P7 vs. P15), *p* < 0.001 (P9 vs. P12), *p* < 0.001 (P9 vs. P15), and *p* < 0.001 (P12 vs. P15).

It has been shown that FNDC3B mRNA is present in the lung of neonatal mice and increases during postnatal development but decreases in adulthood (Kishimoto et al. 2011). However, the expression of FNDC3B mRNA in the developing brain has not been investigated. To examine whether FNDC3B is expressed in PCs during postnatal development, we performed FISH on cerebellar sections at P7, P9, P12, and P15 in mice (Figure 2A–D). We found that FNDC3B is expressed in almost all cells in the PC layer, the external and internal granular layers, and the molecular layer during cerebellar development. To quantify FNDC3B mRNA levels in PCs, we used a probe for GAD67, which is expressed in PCs and basket cells in the PC layer, and discriminated the two by the soma diameter (≈20 μm for PCs and ≈10 μm for basket cells). FNDC3B mRNA signals within PCs were highest at P9 and decreased from P9 to P15 (Figure 2E), suggesting that FNDC3B is expressed in PCs during CF synapse elimination.

### 3.3 CF Synapse Elimination and CF Translocation to PC Dendrites Are Delayed in PC‐Specific FNDC3B‐cKO Mice

**FIGURE 3:**
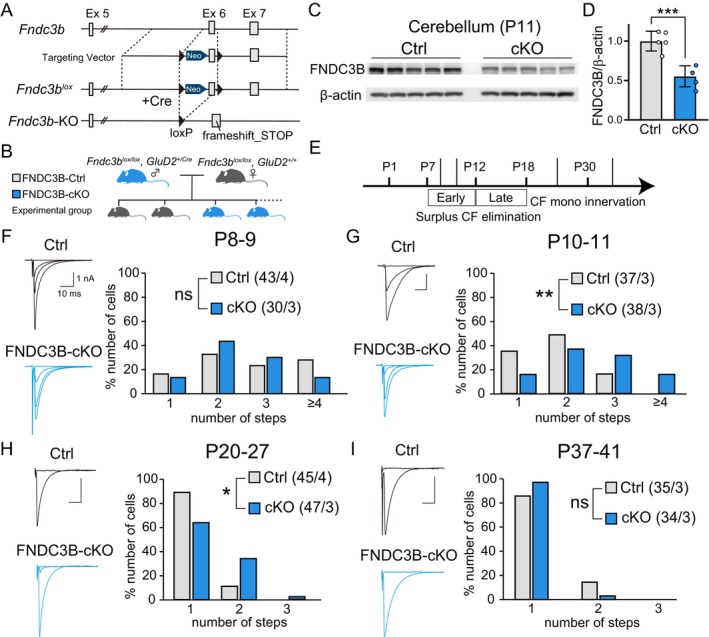
CF synapse elimination is impaired from P10 but restored at around P40 in FNDC3B‐cKO mice. (A) Schema showing the *Fndc3b* genomic DNA (*Fndc3b*), the targeting vector, the targeted genome with loxP sites flanking Exon6 (*Fndc3b* ^lox^), and the *Fndc3b*‐KO genome after Cre recombination (*Fndc3b*‐KO). (B) Breeding scheme. Crossing *Fndc3b* ^*lox/lox*^, *GluD2* ^*+/Cre*^ (FNDC3B‐cKO) male with *Fndc3b* ^*lox/lox*^, *GluD2* ^*+/+*^ (FNDC3B‐Ctrl) female mice to yield the experimental group. (C, D) Immunoblotting data (C) and quantification (D) of relative FNDC3B protein (~133kD) levels in cerebellar homogenates from Ctrl and cKO mice at P11. FNDC3B signals were normalized to β‐actin, and values are expressed relative to the mean of the Ctrl group (each *n* = 5, Student's *t* test, *p* < 0.001). (E) Time course of CF‐PC synapse development showing the time points of electrophysiological experiments for F to I. (F–I) CF innervation of PCs in Ctrl (blue) and cKO (light grey) mice during P8–9 (F), P10–11 (G), P20–27 (H), and P37‐P41 (I). (left) Representative traces of CF‐EPSCs. Scale bars, 10 ms and 1 nA. Holding potential, −10 mV. (right) Frequency distribution histograms for the number of CFs innervating each PC. Mann–Whitney *U* test, *p* = 0.397 (F), *p* = 0.002 (G), *p* = 0.014 (H), and *p* = 0.099 (I). Sample numbers of PCs/mice are shown in parentheses.

To check whether genetic deletion of FNDC3B in PCs affected CF synapse elimination, we generated PC‐specific FNDC3B conditional knockout (FNDC3B‐cKO) mice (Figure 3A, B). To verify FNDC3B deletion at the protein level, we conducted western blotting on cerebellar homogenates at P11 (Figure 3C). Quantification of the relative FNDC3B signal revealed a significant reduction in FNDC3B‐cKO mice compared with Ctrl mice (Figure 3D). Then, we performed the electrophysiological analysis to examine CF innervation of PCs at several stages of postnatal development, sampling PCs across cerebellar lobules in an unbiased manner (Figure 3E–I, Table 1). We found no significant difference in the number of CFs innervating individual PCs between FNDC3B‐cKO and control mice at P8–9 (Figure 3F). The degree of multiple CF innervation was significantly higher in FNDC3B‐cKO mice than in control mice at P10–11 and during P20 to P27 (Figure 3G,H), indicating that the early phase of CF elimination was impaired in FNDC3B‐cKO mice (Figure 3F–H). However, the degree of multiple CF innervation was the same between control and FNDC3B‐cKO mice during P37 to P41 (Figure 3I), indicating that CF mono innervation was achieved by around P40 in the absence of FNDC3B. The basic properties of CF‐EPSCs, including the amplitude, rise time, decay time, and paired pulse ratio (PPR, at 50 ms interval), were not different between control and FNDC3B‐cKO mice for CFs in mono‐innervated PCs (CF‐mono), and the strongest CFs (CF‐multi‐S) and weaker CFs (CF‐multi‐W) in multiply innervated PCs throughout postnatal development from P8 to P27 (Table 2). Moreover, the total amplitude and the disparity (i.e., disparity ratio and disparity index) of CF‐EPSCs in individual PCs were similar between control and FNDC3B‐cKO mice from P8 to P27 (Table 3). These results collectively suggest that FNDC3B promotes CF synapse elimination transiently in the early phase but does not influence basic properties of CF to PC synaptic transmission or selective strengthening of a single “winner” CF in each PC.

**TABLE 1:** Numbers of recorded PCs across cerebellar lobules in Ctrl and FNDC3B‐cKO mice.

Lobule	I/II	III	IV/V	VI/VII	VIII	IX/X
Ctrl (P8–9)	10	10	15	2	2	4
FNDC3B‐cKO (P8–9)	6	6	10	1	4	3
Ctrl (P10–11)	9	9	7	6	2	4
FNDC3B‐cKO (P10–11)	10	11	10	3	1	3
Ctrl (P20–27)	3	9	10	11	6	6
FNDC3B‐cKO (P20–27)	2	8	10	11	8	8
Ctrl (P37–41)	4	6	7	8	4	6
FNDC3B‐cKO (P37–41)	4	7	5	10	4	4

**TABLE 2:** Electrophysiological parameters of CF‐EPSCs in Ctrl and FNDC3B‐cKO mice.

	CF group	Amplitude (nA)	10%–90% rise time (ms)	Decay time constant (ms)	PPR (50 ms)	*n*
Ctrl (P8–9)	CF‐mono	4.14 ± 0.84	0.65 ± 0.07	6.53 ± 1.21	0.45 ± 0.08	4
	CF‐multi‐S	2.07 ± 0.37	0.71 ± 0.06	5.43 ± 0.39	0.51 ± 0.03	17
	CF‐multi‐W	0.95 ± 0.18	0.72 ± 0.07	5.3 ± 0.48	0.73 ± 0.16	24
FNDC3B‐cKO (P8–9)	CF‐mono	2.02 ± 0.84	0.64 ± 0.11	4.99 ± 0.46	0.7 ± 0.03	2
	CF‐multi‐S	2.2 ± 0.35	0.76 ± 0.08	6.38 ± 0.74	0.58 ± 0.06	15
	CF‐multi‐W	0.99 ± 0.17	0.63 ± 0.04	4.81 ± 0.47	0.69 ± 0.09	24
Ctrl (P10–11)	CF‐mono	3.46 ± 0.6	0.5 ± 0.02	3.94 ± 0.54	0.53 ± 0.04	9
	CF‐multi‐S	2.65 ± 0.31	0.51 ± 0.01	3.72 ± 0.35	0.51 ± 0.03	16
	CF‐multi‐W	1.23 ± 0.19	0.48 ± 0.02	2.82 ± 0.4	0.49 ± 0.03	17
FNDC3B‐cKO (P10–11)	CF‐mono	3.67 ± 0.3	0.47 ± 0.03	4.47 ± 0.54	0.62 ± 0.05	5
	CF‐multi‐S	1.84 ± 0.19	0.49 ± 0.02	4.35 ± 0.33	0.58 ± 0.02	22
	CF‐multi‐W	1.16 ± 0.13	0.54 ± 0.03	4.7 ± 0.4	0.58 ± 0.02	30
Ctrl (P20–27)	CF‐mono	2.43 ± 0.11	0.48 ± 0.01	5.56 ± 0.22	0.73 ± 0.01	30
	CF‐multi‐S	2.47 ± 0.03	0.46 ± 0.06	4.63 ± 0.24	0.76 ± 0.05	2
	CF‐multi‐W	0.67 ± 0.24	0.44 ± 0.05	2.33 ± 0.68	0.6 ± 0.09	3
FNDC3B‐cKO (P20–27)	CF‐mono	2.73 ± 0.29	0.49 ± 0.03	6.53 ± 0.61	0.73 ± 0.02	15
	CF‐multi‐S	2.46 ± 0.66	0.53 ± 0.11	5.13 ± 1.26	0.75 ± 0.09	12
	CF‐multi‐W	0.83 ± 0.32	0.53 ± 0.08	4.71 ± 0.74	0.68 ± 0.03	8

**TABLE 3:** Total amplitude, disparity ratio, and disparity index of CF‐EPSCs in Ctrl and FNDC3B‐cKO mice.

	Total amplitude (nA)	Disparity ratio	Disparity index
Ctrl (P8–9)	3.82 ± 0.58 (*n* = 19)	0.63 ± 0.05 (*n* = 15)	0.34 ± 0.06 (*n* = 15)
FNDC3B‐cKO (P8–9)	3.38 ± 0.54 (*n* = 9)	0.54 ± 0.06 (*n* = 13)	0.48 ± 0.08 (*n* = 13)
Ctrl (P10–11)	3.27 ± 0.39 (*n* = 24)	0.59 ± 0.06 (*n* = 11)	0.39 ± 0.07 (*n* = 11)
FNDC3B‐cKO (P10–11)	3.85 ± 0.47 (*n* = 21)	0.55 ± 0.04 (*n* = 12)	0.44 ± 0.06 (*n* = 12)
Ctrl (P20–27)	2.46 ± 0.11 (*n* = 32)	0.17 ± 0.09 (*n* = 2)	1.02 ± 0.18 (*n* = 2)
FNDC3B‐cKO (P20–27)	2.69 ± 0.19 (*n* = 24)	0.3 ± 0.24 (*n* = 7)	0.89 ± 0.35 (*n* = 7)

**FIGURE 4:**
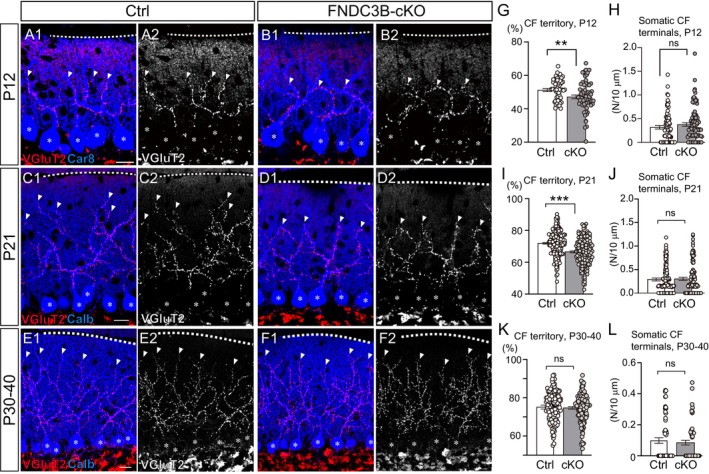
CF translocation is transiently impaired at P12 and P21 but recovered during P30–40 in FNDC3B‐cKO mice. (A–F) Confocal images of Ctrl and cKO cerebella showing double immunofluorescence for Car8/Calb (blue) and VGluT2 (red/white) at P12 (A, B), P21 (C, D), and during P30–40 (E, F). Asterisks indicate PC somata, and arrowheads denote the tips of CFs on the PC dendrites. Scale bar, 20 μm. (G, I, K) Bar graphs showing the relative height of VGluT2‐labeled CF terminals normalized by the molecular layer thickness at P12, P21, P30–40 (Ctrl, *n* = 60, 226, 132 PCs; cKO, *n* = 63, 182, 124 PCs.) (H, J, L) Bar graphs quantifying the number of VGluT2‐positive CF terminals per 10 μm along the PC soma at P12, P21, P30–40 (Ctrl, *n* = 77, 154, 60 PCs; cKO, *n* = 93, 134, 60 PCs). 3 mice for each group. Mann–Whitney *U* test, *p* = 0.0076 (G), *p* = 0.209 (H), *p* < 0.001 (I), *p* = 0.816 (J), *p* = 0.419 (K), and *p* = 0.649 (L).

To investigate whether the lack of FNDC3B in PCs affects CF translocation along PC dendrites, we immunostained VGluT2, a CF terminal marker, and Car8/Calb, a marker for PC soma and dendrites, at P12, at P21, and from P30 to P40. We measured the height of VGluT2 signals relative to that of Car8/Calb signals, representing the extent of CF innervation over PC dendritic arbors. We found that the relative height in FNDC3B‐cKO PCs was significantly lower than in control PCs at P12 and P21 (Figure 4A–D,G,I). However, the values were not significantly different between the two genotypes from P30 to P40 (Figure 4E,F,K). These results suggest that FNDC3B facilitates the extension of CFs over PC dendrites transiently until around P21 without a persistent reduction in the degree of CF translocation after around P30. Although our electrophysiological data show the transient impairment of CF synapse elimination in FNDC3B‐cKO mice from around P10 to P27 (Figure 3G,H), the density of VGluT2 puncta representing CF terminals on the PC soma was not significantly different between control and FNDC3B‐cKO mice at P12, at P21, and from P30 to P40 (Figure 4H,J,L).

### 3.4 Excitatory PF Synapses and Inhibitory Synapses on PCs Are Normal in FNDC3B‐cKO Mice

**FIGURE 5:**
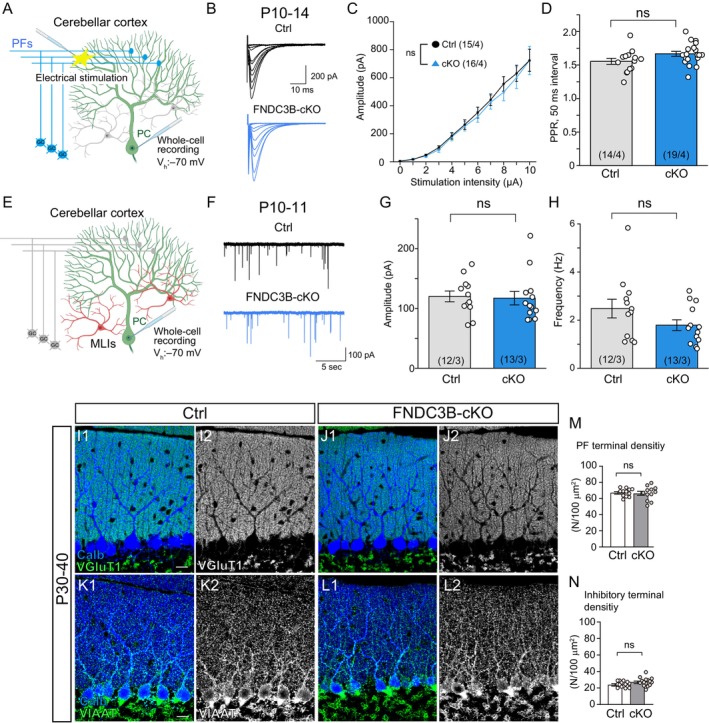
PF‐PC excitatory synapses and MLIs‐PC inhibitory synapses are not altered in FNDC3B‐cKO mice. (A) Schema showing whole‐cell recordings of PF‐EPSCs from a PC. Holding potential, −70 mV. (B) PF‐EPSC traces from a Ctrl (grey) and a cKO (blue) PC. Scale bars, 10 ms and 200 pA. (C) Relationships between the stimulation intensity and the amplitude of PF‐EPSCs. Two‐way repeated measures ANOVA, Genotype; *p* = 0.21, intensity; *p* < 0.0001, Genotype and intensity; *p* = 0.548 (D) Paired‐pulse ratio (PPR) of PF‐EPSCs at 50 ms interval at P10–14. Student's *t* test, *p* = 0.059 (D). (E) Schema showing whole‐cell recordings of mIPSCs from a PC. Holding potential, −70 mV. (F) mIPSC traces recorded from a Ctrl (grey) and a cKO (blue) PC. Scale bars, 5 s and 100 pA. (G, H) Amplitude (G) and frequency (H) of mIPSCs at P10–11. Mann–Whitney *U* test, *p* = 0.538 (G) and *p* = 0.225 (H). Sample numbers of PCs/mice are shown in parentheses. (I–L) Confocal images of Ctrl and cKO cerebella showing Calb (blue) with VGluT1 (green/white, I, J) or VIAAT (green/white, K, L) during P30–40. Scale bar, 20 μm. (M, N) Density of VGluT1‐positive PF terminals in Ctrl (*n* = 12 areas) and cKO (*n* = 12 areas) mice (M), and VIAAT‐positive inhibitory terminals in Ctrl (*n* = 15 areas) and cKO (*n* = 15 areas) mice (N) during P30–40. 3 mice for each group. Student's *t* test, *p* = 0.733 (M) and *p* = 0.052 (N). Error bars represent ± SEM.

Establishment of excitatory PF synapses and maturation of inhibitory synapses on PCs during postnatal development are crucial factors for CF synapse elimination (Hashimoto et al. 2001; Hashimoto, Yoshida, et al. 2009; Ichikawa et al. 2002; Nakayama et al. 2012; Lai et al. 2025). To test whether the lack of FNDC3B in PCs affected excitatory synaptic transmission from PFs and inhibitory synaptic transmission in PCs, we recorded PF‐mediated EPSCs (Miyazaki et al. 2004; Zhang et al. 2025) and mIPSCs (Yamasaki et al. 2006; Zhang et al. 2025) from PCs in control and FNDC3B‐cKO mice during development (Figure 5A–H). We found no significant differences between the two genotypes in the input–output relationship and paired‐pulse ratio (PPR) at 50 ms interval of PF‐EPSCs during P10–14 (Figure 5A–D) and the frequency and amplitude of mIPSCs at P10–11 (Figure 5E–H). We also investigated the distribution of excitatory PF and inhibitory synapses on PCs by immunostaining the PF marker VGluT1 and the inhibitory synaptic terminal marker VIAAT in the cerebellum from P30 to P40 (Figure 5I–L). We found that the densities of PF terminals and inhibitory terminals in the molecular layer were not significantly different between wild‐type and FNDC3B‐cKO mice (Figure 5M,N). These results strongly suggest that both excitatory PF and inhibitory synapses on PCs are normal in FNDC3B‐cKO mice.

### 3.5 FNDC3B May Contribute to CF Synapse Elimination Through a Pathway Distinct From P/Q‐VDCC‐Dependent Signaling

**FIGURE 6:**
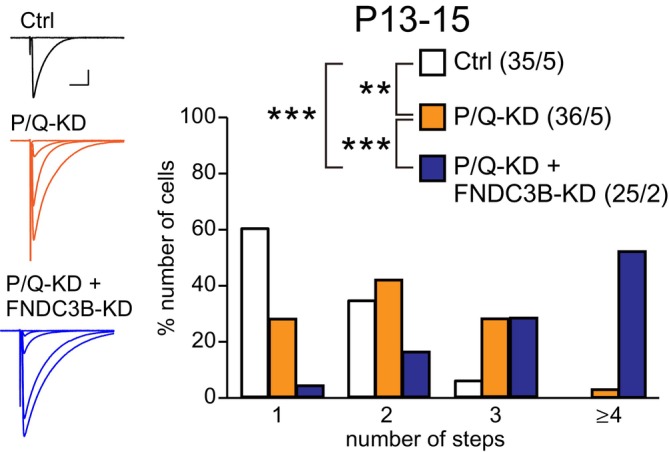
Double‐KD of P/Q‐VDCC and FNDC3B results in a more severe impairment of CF synapse elimination than single KD of P/Q‐VDCC. Representative CF‐EPSC traces (left) and frequency distribution histograms indicating the number of CFs innervating each PC (right) during P13–15 for untransfected control PCs (Ctrl, white square), PCs with P/Q‐VDCC‐KD (P/Q‐KD, orange square), PCs with double KD of P/Q‐VDCC and FNDC3B (P/Q‐KD + FNDC3B‐KD PCs, blue square). Scale bars, 1 nA and 10 ms. Holding potential, −10 mV. The sample numbers of PCs/mice are shown in parentheses. Kruskal–Wallis test, *p* < 0.001, followed by Steel–Dwass test, *p* = 0.004 (Ctrl vs. P/Q‐KD), *p* < 0.001 (Ctrl vs. P/Q‐KD + FNDC3B‐KD), and *p* < 0.001 (P/Q‐KD vs. P/Q‐KD + FNDC3B‐KD).

Our previous studies demonstrate that the early phase of CF elimination is critically dependent on P/Q‐VDCC‐mediated Ca^2+^ influx into PCs (Miyazaki et al. 2004; Hashimoto et al. 2011). Since the early phase of CF elimination was impaired in FNDC3B‐cKO mice (Figure 3G), FNDC3B may contribute to CF synapse elimination along the P/Q‐VDCC‐mediated pathway in PCs. To check this possibility, we compared the effect of double KD of FNDC3B and P/Q‐VDCC and that of single KD of P/Q‐VDCC (Figure 6). We prepared a lentivirus for P/Q‐VDCC‐KD and mOrange expression and injected this lentivirus alone (for single KD of P/Q‐VDCC) or together with the lentivirus for FNDC3B‐KD and GFP expression (for double KD of P/Q‐VDCC and FNDC3B) into the cerebellum of wild‐type mice at P1–2. We performed electrophysiological examination from P13 to P15 to compare CF innervation between PCs with single KD of P/Q‐VDCC (P/Q‐KD) and those with double KD of P/Q‐VDCC and FNDC3B (P/Q‐KD + FNDC3B‐KD). We found that the degree of multiple CF innervation in PCs with single P/Q‐VDCC‐KD was significantly higher than in untransfected control PCs (Figure 6). Notably, the degree of multiple CF innervation in PCs with double P/Q‐VDCC and FNDC3B‐KD was considerably higher than that in PCs with single P/Q‐VDCC‐KD (Figure 6), demonstrating that P/Q‐VDCC‐KD did not occlude the effect of FNDC3B‐KD. This result indicates that FNDC3B contributes to CF synapse elimination through a pathway at least partly distinct from the P/Q‐VDCC pathway.

Taken all together, the results of the present study indicate that FNDC3B in PCs promotes CF synapse elimination and the dendritic translocation of the strongest CF, presumably through a P/Q‐VDCC‐independent pathway, but has no detectable effect on excitatory PF and inhibitory synapses on PCs.

## 4 Discussion

### 4.1 FNDC3B Facilitates CF Synapse Elimination and Dendritic CF Translocation

In the present study, we demonstrated that deletion of FNDC3B in developing PCs impaired CF synapse elimination from around P10 and reduced the degree of CF dendritic translocation at P21. In the FNDC3B‐KD experiments, an apparent increase in the degree of multiple CF innervation was detected at P8–10, whereas a comparable change was not observed in FNDC3B‐cKO mice at P8–9. Because GFP + and GFP − PCs were sampled partly from different lobules in FNDC3B‐KD experiments, we cannot exclude the possibility that this sampling bias might have influenced the result. Therefore, our conclusion that FNDC3B deletion in PCs impaired CF synapse elimination from around P10 is based on the dataset from FNDC3B‐cKO mice, in which PCs were sampled in an unbiased manner across lobules (Table 1). However, these phenotypes were transient, lasting until approximately P25, and were not observed in the more mature cerebellum. In contrast, the lack of FNDC3B in PCs did not affect the basic properties of CF‐PC synaptic transmission (Table 2), and the selection of a single winner CF in each PC, as evidenced by the lack of significant differences in the disparity index and disparity ratio between the genotypes (Table 3), although the absolute ranges of these parameters differ somewhat from those reported previously (Hashimoto and Kano 2003; Hashimoto, Ichikawa, et al. 2009; Hashimoto, Yoshida, et al. 2009; Nakayama et al. 2012). While the reasons for this difference are not clear, it may reflect a possible difference in cerebellar regions or lobules from which PCs were recorded. Besides, the deletion of FNDC3B in PCs did not influence the development of excitatory PF synapses and GABAergic inhibitory synapses on PCs (Figure 5). These results suggest that FNDC3B in PCs facilitates the early phase of CF elimination and dendritic translocation of the winner CF until around P21. Although multiple CF innervation persists around P21 in FNDC3B‐cKO mice, our current data cannot determine whether FNDC3B directly contributes to the late phase of CF elimination or whether the phenotype reflects a delay in the overall CF synapse elimination process caused by impaired early phase despite normal late phase of CF elimination.

It has been reported that the deletion of P/Q‐VDCC from PCs severely impairs the selection of a single winner CF (Hashimoto et al. 2011), its translocation to PC dendrites (Hashimoto et al. 2011), and the early and late phases of CF elimination (Miyazaki et al. 2004; Hashimoto et al. 2011; Mikuni et al. 2013; Kawata et al. 2014), and causes a reciprocal expansion of PF innervation on PC dendrites (Miyazaki et al. 2004, 2012) and enhanced PF‐PC synaptic transmission (Miyazaki et al. 2012). These multiple defects in CF and PF synaptic wiring to PCs are thought to be primarily caused by the reduced Ca^2+^ influx to PCs from CF inputs (Hashimoto et al. 2011). While the early phase of CF elimination and dendritic translocation of the winner CF were impaired in FNDC3B‐cKO mice, which are analogous to those seen in PC‐specific conditional P/Q‐VDCC KO (P/Q‐VDCC‐cKO) mice (Hashimoto et al. 2011), the phenotypes in FNDC3B‐cKO mice are transient and much milder than those of P/Q‐VDCC‐cKO mice. Notably, double KD of P/Q‐VDCC and FNDC3B in PCs resulted in a more severe impairment of CF synapse elimination than single KD of P/Q‐VDCC, suggesting that FNDC3B contributes to CF synapse elimination independently of P/Q‐VDCC. Thus, FNDC3B in PCs may facilitate the early phase of CF elimination and dendritic translocation of the winner CF through a mechanism that is at least in part independent of canonical P/Q‐VDCC‐dependent signaling.

Relatively little is known about molecules involved in the early phase of CF elimination, both those acting downstream of P/Q‐VDCC and those operating independently of P/Q‐VDCC. Previous studies show that the immediate early gene *Arc/Arg3.1* (Arc) is activated by the P/Q‐VDCC‐mediated Ca^2+^ rise in PCs and promotes the late phase of CF elimination (Mikuni et al. 2013; Kawata et al. 2014). However, the KD of Arc in PCs did not affect the selection of a single winner CF, its dendritic translocation, and the early phase of CF elimination (Mikuni et al. 2013). Thus, Arc is considered to contribute specifically to the late phase of CF elimination along the P/Q‐VDCC‐triggered signaling cascades. Our recent study shows that the transcription factor ZFP64 promotes the late phase of CF elimination, dendritic translocation of a winner CF, and the reciprocal enhancement of PF‐PC synaptic transmission, presumably downstream of P/Q‐VDCC (Zhang et al. 2025). However, these phenotypes were milder than those of P/Q‐VDCC‐cKO mice and restored in maturity (Zhang et al. 2025), suggesting that molecular cascades other than those activated by ZFP64 must constitute the canonical pathways downstream of P/Q‐VDCC. Thus, FNDC3B may act in a distinct pathway that operates in parallel with these P/Q‐VDCC‐dependent signaling.

As for other molecules involved in the early phase of CF elimination, C1q‐like molecule 1 (C1ql1) derived from CFs acting anterogradely on cell adhesion G‐protein‐coupled receptor 3 (Bai3) in PCs is reported to promote CF elimination from P7 and the strengthening and maintenance of the strongest CF (Kakegawa et al. 2015). In contrast, Semaphorin 3A (Sema3A) is shown to be secreted from PCs and acts retrogradely to its receptor Plexin A4 (PlxnA4) on CFs from around P8 to around P18 (Uesaka et al. 2014). Because the loss of either of these signals significantly reduces the strength of CF to PC synaptic transmission in adulthood, which was not seen in FNDC3B‐cKO mice, FNDC3B is unlikely to act on these pathways. On the other hand, reduced GABAergic inhibition on PCs from around P7 to around P15 resulted in impaired CF synapse elimination from P10 in GAD67 heterozygous KO mice (Nakayama et al. 2012) and neuroligin 2 KO mice (Lai et al. 2025). However, because GABAergic inhibition on PCs at P10–11 was not altered in FNDC3B‐cKO mice, FNDC3B in PCs is not necessary, if any, for the development of inhibitory synapses. Thus, the impaired CF synapse elimination in FNDC3B‐cKO mice is independent of alterations in GABAergic inhibition on PCs.

A distinctive feature of the phenotypes in FNDC3B‐cKO mice is that the impaired CF synapse elimination and reduced dendritic CF translocation were transient until around P20 to P30, and they did not persist into adulthood. Because the expression levels of FNDC3B mRNA in PCs peak at P9, the lack of FNDC3B may affect the processes of CF synapse elimination during a limited period from around P10 and may be compensated in the mature cerebellum. These properties indicate that FNDC3B functions as a modulatory factor that refines CF synapse elimination, rather than as an indispensable core component. For example, FNDC3A, a paralog of FNDC3B sharing half of the amino acid sequence with FNDC3B, is localized in the ER (Fucci et al. 2020), might, in principle, compensate for the lack of FNDC3B, although we currently have no direct evidence for such compensation. Analogous transient impairments in CF synapse elimination with restoration of normal CF mono‐innervation in maturity were observed in Ca^2+^/calmodulin‐dependent protein kinase IIα (CaMKIIα)‐KO mice (Hansel et al. 2006), mutant mice deficient in myosin Va (*Myo5a*) (Takagishi et al. 2007), serotonin 3A receptor (*Htr3a*/5‐HT_3A_) KO mice (Oostland et al. 2013), mice with an arginine to cysteine substitution of amino acid residue 451 (R451C) in *Nlgn3* (Lai et al. 2021), and mice with PC‐specific KD of ZFP64 (Zhang et al. 2025). It remains unclear why seemingly similar phenotypes are observed across these genetically modified mouse lines. These genes likely do not encode core components of the main pathways for CF synapse elimination, but rather encode molecules that modulate the main pathways. Since the core pathways remain functional, any transient defects in CF synapse elimination during development may be compensated over time, resulting in normal circuit maturation in adulthood. The precise molecular mechanisms underlying such compensation, including a potential contribution by FNDC3A and other redundant molecules, remain to be elucidated in future studies.

### 4.2 Possible Functions of FNDC3B

Although the protein structure of FNDC3B and in vitro studies suggest that FNDC3B is a resident protein of the ER/Golgi apparatus, the precise function of FNDC3B remains largely unclear. Since ER proteins are involved in various cellular functions such as protein folding, protein transport, lipid synthesis, and calcium release (Schwarz and Blower 2016) FNDC3B may contribute to these ER functions in PCs. Protein folding and transport generally control protein maturation and homeostasis, influencing trafficking, expression levels, and functions of various proteins in PCs. Calcium release from the ER could affect the activity of calcium‐dependent kinases, including CaMKIIα and PKCγ, known to be involved in CF synapse elimination. On the other hand, a previous study has shown that FNDC3B directly interacts with the signal transducer and activator of transcription 3 (STAT3) and inhibits its activity in melanoma cells (Katoh et al. 2015). STAT3 in the nervous system has multiple roles, including promoting axonal growth and maintenance in sensory and motor neurons after lesions (Schweizer et al. 2002; Lee et al. 2004), neurite outgrowth and synapse formation after oxygen–glucose deprivation (Chen et al. 2016), and neuronal survival (Yadav et al. 2005). Deletion of STAT3 from PCs caused an increase in mEPSC amplitude and a decrease in mIPSC frequency with transcriptional changes (Han et al. 2021), suggesting that STAT3 plays a role in regulating synaptic transmission in PCs. However, whether CF synapse elimination is affected by the loss of STAT3 has yet to be investigated. Moreover, FNDC3B has been implicated in cell adhesion via Integrins and in the regulation of TGF‐β receptor trafficking and signaling in non‐neuronal systems (Tominaga et al. 2004; Cai et al. 2012). Also, the FNDC3 ortholog *mtgo* in Drosophila is required for neuromuscular junction branching and synaptic physiology, supporting a conserved role of FNDC3 family proteins in shaping neuronal connectivity (Syed et al. 2019). It raises the possibility that FNDC3B in PCs may modulate CF synapse elimination through cell‐adhesion or surface‐receptor–related mechanisms. In cancer research, increasing attention has recently been directed toward the roles of circular (circ) RNAs derived from FNDC3B (Sun et al. 2023). These circRNAs have been reported to be involved in several cancer cells, possibly through the alteration of gene expression, acting as a biomarker (Pan et al. 2020; Wang et al. 2022; Chen et al. 2023) as well as in the mammalian brain development and function (Rybak‐Wolf et al. 2014; Dong et al. 2023). Therefore, not only proteins but also mRNAs and circRNAs might be altered in their expression and function in PCs of FNDC3B‐cKO mice. Further studies are required to elucidate how FNDC3B in PCs facilitates CF synapse elimination and dendritic CF translocation.

## Author Contributions

**Céline Louise Mercier:** conceptualization, data curation, formal analysis, investigation, writing – original draft. **Takaki Watanabe:** conceptualization, data curation, formal analysis, investigation, supervision, writing – original draft. **Yuto Okuno:** conceptualization, data curation, formal analysis, investigation, writing – original draft. **Kyoko Matsuyama:** formal analysis, investigation. **Kyoko Kushibe:** formal analysis, investigation. **Henry Denny:** formal analysis, investigation. **Taisuke Miyazaki:** data curation, formal analysis, investigation. **Miwako Yamasaki:** data curation, formal analysis, investigation. **Meiko Kawamura:** resources. **Manabu Abe:** resources. **Kenji Sakimura:** resources. **Masahiko Watanabe:** formal analysis, investigation. **Naofumi Uesaka:** conceptualization, writing – original draft. **Masanobu Kano:** conceptualization, funding acquisition, supervision, writing – review and editing.

## Funding

This work was supported by Japan Society for the Promotion of Science, 18H04012, 20H05915, 21H04785.

## Conflicts of Interest

The authors declare no conflicts of interest.

## Data Availability

The data that support the findings of this study are available from the corresponding authors upon reasonable request.
